# Acute hepatitis of unknown aetiology in children: a clinical update on the recent outbreak with mechanistic insights

**DOI:** 10.1093/cei/uxad023

**Published:** 2023-02-15

**Authors:** Sunitha Vimalesvaran, Anita Verma, Eirini Kyrana, Maesha Deheragoda, Anil Dhawan

**Affiliations:** Paediatric Liver, Gastroenterology and Nutrition Centre, King’s College Hospital NHS Trust, London, UK; Department of Infection Sciences, King’s College Hospital, London, UK; Paediatric Liver, Gastroenterology and Nutrition Centre, King’s College Hospital NHS Trust, London, UK; Paediatric Liver, Gastroenterology and Nutrition Centre, King’s College Hospital NHS Trust, London, UK; Paediatric Liver, Gastroenterology and Nutrition Centre, King’s College Hospital NHS Trust, London, UK

**Keywords:** acute hepatitis, acute liver failure, paediatric

## Abstract

Since April 2022, over 1000 children across 35 countries have developed episodes of acute hepatitis of unknown origin. At King’s College Hospital, a total of 65 children were referred with acute hepatitis of unknown etiology, with 10 of these children presenting with acute liver dysfunction leading to acute liver failure. Multiple hypotheses have been proposed and continue to be investigated worldwide. In this review, we explore the current understanding of potential aetiologies for this outbreak. We further characterize the proposed immunological mechanisms of liver injury in these cases.

## Teaser

Health alerts have been triggered across multiple countries with regards to reports of severe adenovirus-associated acute hepatitis in previously well children. Multiple hypotheses have been proposed and continue to be investigated worldwide. In this review, we discuss the potential aetiologies and underlying mechanisms of liver injury of this outbreak.

## Graphical Abstract

**Graphical Abstract:**
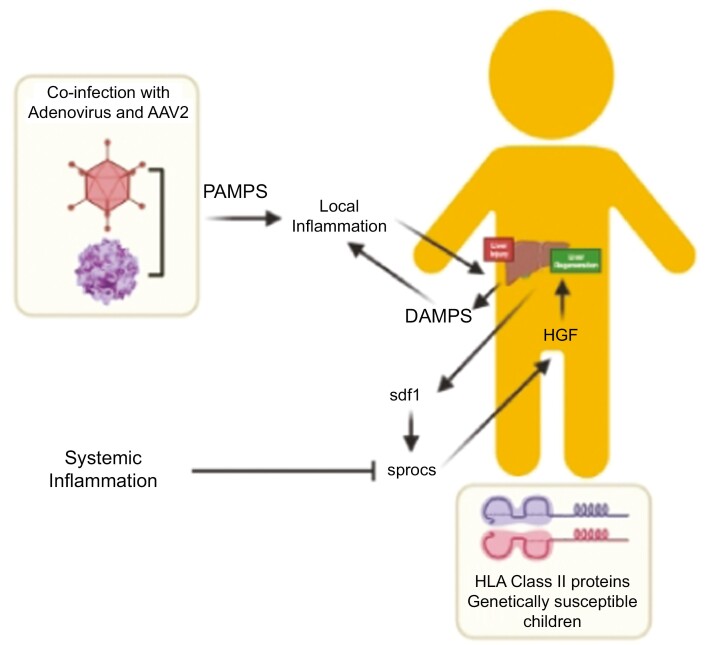

Since April 2022, in the UK and some other countries, there has been an outbreak of children with acute hepatitis of unknown aetiology. By 8 July 2022, the number of probable cases of acute hepatitis in children had risen to 1010, across 35 countries. Of these, 46 children (5%) had liver transplants and 22 deaths (2%) were reported by the World Health Organisation [1]. The highest number of cases (484 cases; 48% of global cases) were reported from Europe (21 countries), with the United Kingdom (UK) contributing to 274 cases (27% of global cases) as of 4 July 2022 [2]. In the UK, 15 children required liver transplantation and no deaths have been reported. At King’s College Hospital, a total of 65 children were referred with acute hepatitis of unknown aetiology, with 10 of these children presenting with acute liver dysfunction leading to acute liver failure (PALF). Seven of these children went on to receive a liver transplant at our centre.

The affected children were previously well and generally under the age of 5 years (median age 3 years, interquartile range 2–5 years). They demonstrated markedly raised liver biochemistry with aspartate aminotransferase (AST) or alanine aminotransferase (ALT) over 500 U/L at presentation (agreed case definition by UK Health Security Agency (UKHSA). The most common presenting symptoms were jaundice and gastrointestinal symptoms such as vomiting and diarrhoea [2]. Cases were generally distributed sporadically, without epidemiological association or commonality in terms of travel history.

Multiple hypotheses have been proposed and continue to be investigated worldwide [3]. There remains limited evidence to *definitively* establish a causal relationship between this outbreak of acute hepatitis and any potential aetiology. The leading hypothesis continues to involve human adenovirus (HAdV), as the most commonly identified potential pathogen in the UK cohort. Ninety-four percent of patients underwent testing for HAdV in the UK, with 170 cases (65.9%) testing positive for HAdV, necessitating treatment with antivirals like Cidofovir in children with high levels of adenoviraemia. We showed that Cidofovir resulted in a reduction in viral load and non-recurrence of hepatitis in the allograft, with only transient nephrotoxicity as a side effect in our cohort of children presenting with PALF [4]. Partial hexon gene sequencing further identified type 41F as the key HAdV subtype detected in these cases. This hypothesis was further supported by the marked 4-fold increase in the UK community prevalence of HAdV among children under the age of 4 since November 2021, compared to the pre-pandemic and pandemic period [5], likely secondary to the impact of the COVID-19 pandemic necessitating isolation on child mixing and infection patterns. This recent outbreak also corresponded to the relaxation of social distancing regulations in 2022 and waning of maternal immunity in children born during the COVID-19 pandemic [6]. However, HAdV-41F is known to only cause a self-limiting illness with gastrointestinal symptoms and/or respiratory symptoms in immunocompetent hosts and is rarely reported to cause liver injury in children with healthy immunity. There appears to be a “threshold effect” with HAdV, which prevents the virus from reaching the hepatocyte unless adenovirus capture by liver Kupffer cells is saturated. HAdV in itself does not usually infect hepatocytes and even if it does, there should be a finding that Kupffer cells are saturated with HAdV [7]. We and others have not demonstrated immunohistochemical evidence of HAdV or electron microscopic evidence of viral particles in liver biopsies or explant livers [8].

More recently, large amounts of adeno-associated virus type 2 (AAV-2) on metagenomic and PCR analyses of blood and liver tissues have been identified in a subset of the UK cohort. Highly abundant amounts of AAV-2 were found in the five explanted livers and in blood samples of the 11/12 non-transplanted cases, as opposed to infrequent detection in controls [9]. AAV-2 is only able to replicate in the presence of a helper viruses such as HAdV, herpesviruses, or papillomavirus [10]. Therefore, it is perhaps reasonable to assume that the high replication of AAV-2 in these cases was secondary to a contributory infection with HAdV. The lack of viral proteins or virion particles in liver biopsies, however, suggests that it is unlikely that these cases are caused by a direct lytic infection involving either or both viruses.

The mechanism in which AAV-2 is implicated alongside a co-infection with HAdV infection is possibly through an aberrant immune-mediated host response. This is evidenced by cases of hepatitis described in the early trials of AAV-2, when used as a viral vector for gene therapy, with a possible CD8+ cell-mediated response directed against the AAV-2 viral capsid (VP1) [11, 12]. Chapin et al. have also previously shown in children with indeterminate acute liver failure, that there appear to be CD8+ T-cell infiltrates which are clonal, indicating an antigen-driven response [13]. The seven explants from our cohort demonstrated non-specific cholestatic hepatitis, with large areas of confluent hepatocyte necrosis, hepatocyte emperipolesis, multinucleation, and giant cell change. These features have been reported in autoimmune liver disease and hepatitis associated with Coombs’ positive haemolytic anaemia, both of which were excluded in these patients. However, these findings could suggest that an underlying immunological mechanism may be responsible, in these seemingly immunocompetent children. Moving forwards, it would be pivotal to further understand the immune responses in these children.

Genetic susceptibility has also been postulated in this cohort, with HLA typing of 20 Scottish children and 64 controls demonstrating an association of the Class II HLA DRB1*0401 allele in affected cases. Further proteomic data from explanted livers also showed high levels of HLA Class II proteins [14]. Together, this puts forth the hypothesis that hepatitis seen in these children is immune-mediated, occurring in children who are genetically predisposed and potentially triggered by an infection of AAV-2 and HAdV.

Of the 162 cases tested for SARS-CoV-2 in the UK, only 19 cases tested positive at admission or in the preceding 8 weeks prior to admission (11.6%). There was no statistically significant difference in antibody positivity between hepatitis cases and NHS patient controls. It has also been proposed that SARS-CoV-2 persistence in the gut with immunological sensitization from the spike protein superantigen coupled with adenoviral gut infection, may have potentially led to IFNγ release and IFNγ-mediated hepatocyte apoptosis [15]. It is difficult to tease out the contribution of past SARS-CoV-2 infections to the recent outbreak of acute hepatitis of unknown aetiology. The absence of such an outbreak during the pandemic makes SARS-CoV-2 infection an unlikely causative agent.

Whilst there has been a surge of acute hepatitis cases in children, we believe that this may not be a new association between HadV and PALF. In the five years prior to 2022, we saw between three and six cases per year of PALF of unknown aetiology at our centre, compared to the 10 cases reported in the first half of 2022. Between 2017 and 2019, HAdV was detected in 25–75% of these cases, none in years 2020 and 2021 and 100% in 2022. We believe that this may be a new epidemiological phenomenon in the post-pandemic period, of an already well-described disease of PALF of unknown aetiology, albeit with a higher morbidity rate.

It is pivotal that we use this opportunity to better elucidate the pathogenesis and management strategies for PALF of unknown aetiology. One of the key areas of research would be to better characterize the mechanisms of liver injury. Alongside a systemic inflammatory insult, exogenous local triggers including viruses containing pathogen associated molecular patterns (PAMPs) and endogenous local triggers in the form of damage associated molecular patterns (DAMPs) generated from the liver injury itself, are thought to cause ongoing liver inflammation in PALF. This results in the production of stromal cell-derived factor 1 (sdf1) to recruit sinusoidal endothelial cell progenitor cells (sprocs), and subsequently liver regeneration may occur through release of hepatocyte growth factor (HGF). This process may be interrupted further through the overwhelming systemic inflammatory response leading to suppression of the bone marrow and thus inhibition of the release of sprocs [16]. Biorepositories of human samples from patients with PALF, animal models of immune dysregulation, metagenomics, and histopathology could provide important insights into the patterns of immune-mediated injury and potentially aid in the development of targeted therapies for PALF of unknown aetiology. In future, the use of immunosuppression in a subset of PALF children who demonstrate histological changes on biopsy and immunostaining, with markers of immune activation in the setting of genetic predisposition is an attractive therapeutic option for this condition that historically has required liver transplantation more often compared to children where aetiology of PALF is identified [16].

Interestingly there appears to be a sudden halt in new patients referred in the UK and to our centre from August 2022, suggesting an epidemiological phenomenon of the increased occurrence of acute liver dysfunction of indeterminate aetiology with population dynamics reset to pre-pandemic levels. Based on recent evidence, AAV-2 alongside a “helper virus” like HadV appears to have played a role in initiating an immune response in genetically susceptible individuals to cause acute hepatitis and PALF, in some. Further pathological investigation will be important in understanding the immunopathologic damage in the liver of these children. As we continue to understand the aetiology of this current outbreak, it is important to make a concerted global effort to map and identify relevant cases. Epidemiological studies will be of paramount importance in disease prevention.

## Data Availability

Not applicable.

## Abbreviations

AAV-2: adeno-associated virus type 2
ALT: alanine aminotransferase
AST: aspartate aminotransferase
DAMPs: damage associated molecular patterns
HAdV: human adenovirus
PALF: paediatric acute liver failure
PAMPs: pathogen associated molecular patterns
